# Development of Thymic Organoids Heterotopically to Educate and Induce T Lymphocytes

**DOI:** 10.1002/iid3.70229

**Published:** 2025-08-22

**Authors:** Xiuxia Wang, Shun Yu, Yucheng Qiu, Jun Yang, Fei Liu, Xianyu Zhou

**Affiliations:** ^1^ Department of Plastic and Reconstructive Surgery Shanghai Ninth People's Hospital, Shanghai JiaoTong University School of Medicine Shanghai China; ^2^ Department of Burns and Plastic Surgery Affiliated Hospital of Jiangnan University Wuxi China

**Keywords:** hematopoietic stem cells, T lymphocytes, thymic epithelial cells, thymic mesenchymal cells, vascularized composite allotransplantation

## Abstract

**Background:**

Vascularized composite allotransplantation (VCA) is a potential treatment for extensive injuries that replaces defects like‐with‐like, however allografts are immune‐rejectable.

**Methods:**

This study developed in vitro thymic organoids and examined whether donor‐derived HSCs could be educated in vivo into T lymphocytes via central tolerance. TECs, TMCs, and HSCs from C57BL/7 (CD45.2^+^) or SJL/L (CD45.1^+^) mice were labeled with cell surface markers and examined by flow cytometry. Co‐culturing three cell lines in vitro created thymic aggregates. Aggregates transplanted to C57BL/7 (CD45.2^+^) mice′s inguinal regions developed thymic organoids. Immunorejection genes were identified bioinformatically. Western blot, immunofluorescence, and flow cytometry were utilized to measure rejection‐related protein levels and T cell surface markers in thymic organoids to determine T cell inducement and immunomodulation.

**Results:**

In vitro, TECs, TMCs, and HSCs created thymic aggregates, which became thymic organoids after in vivo transplantation and produced CD8^+^ and CD4^+^ Tregs. Bioinformatics showed high correlations between transplanted rejection and IFNG, IL2RG, FCGR3A, and ICAM1 genes. Immunofluorescence and Western blot showed increased protein expression of IFNG, IL2RG, FCGR3A (immunomodulation biomarker), and decreased protein expression of CK8, CK14, and ICAM1 (TEC biomarker) in thymic organoids.

**Conclusion:**

Thymic organoids heterotopically implanted in vivo can promote heterologous HSC‐derived T cell development.

## Graphical abstract

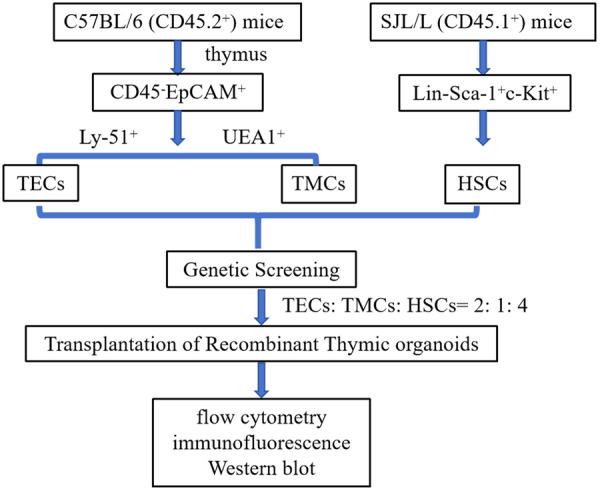

## 1 Introduction

Vascularized composite allotransplantation (VCA) is a promising therapy for patients with severe, complex tissue destruction of limb and face, et al., caused by trauma, burn, and tumor [1]. VCA grafts usually contain tissues from different germ layer development sources, such as skin, muscle, bone, nerve and blood vessel, with strong antigenicity, and the grafts are subject to immune rejection [2, 3]. Cellular therapy aimed at modulating immune rejection has been witnessed in VCA [4].

Stem cells, especially bone marrow hematopoietic stem cells (HSCs), play an important role in immune regulation [5]. In 2008, abundant reports revealed that a combination of immunosuppressant and donor‐derived HSCs transplantation can successfully establish stable and durable immune tolerance in human leucocyte antigen‐mismatched kidney transplantation [6, 7] and liver transplantation [6] patients. Transplantation immune tolerance means that the recipient does not need long‐term immunosuppressive medication to ensure no rejection of the graft and maintain a complete immune response to a third‐party alloantigen [8]. The immune tolerance to VCA transplantation has been successively induced and established in large or small animals through total body irradiation (TBI) combined with multiple immunomodulators/immunosuppressants, and cell therapies such as HSCs or T regulatory cells (Tregs) [9, 10]. Immune tolerance includes central and peripheral tolerance [11]. Peripheral tolerance control has been the primary focus of earlier research on immunological rejection. In the primary lymphoid organs (namely the thymus and bone marrow), central tolerance develops throughout T lymphocytes (T cells) development [11], suggesting that its modulation may address the root etiology of VCA immunological rejection.

HSCs, thymic epithelial cells (TECs) and thymic mesenchymal stem cells (TMSCs) interact to enhance T cells development. To be more specific, after homing to the thymic cortex, HSCs differentiate into immature thymic cells and then interact with major histocompatibility complex (MHC) peptide complexes presented by cortical thymic epithelial cells (cTECs) [12]. They differentiate into cluster of differentiation 4 receptors (CD4)^+^ CD8^+^ T‐lymphocyte (T) cells after positive selection [13], then are mutually recognized with a variety of tissue‐specific and concealed autoantigens synthesized by medullary thymic epithelial cells (mTEC), and eventually mature into functional CD3^+^ CD8^+^ T cells, CD3^+^ CD4^+^ T cells and Tregs after negative selection [14, 15]. Despite not being immune cells, TECs still play a leading role in the induction and self‐recognition process of “central tolerance” of T cells as an important component of thymus tissue. Reportedly, the interaction between TECs and thymic mesenchymal cells (TMCs) is important for establishing a normal thymus microenvironment to T cells development [16]. TMCs greatly influence the survival and maturation of TECs, which in turn coordinates the proliferation and differentiation of T cells.

In this study, we cocultured TECs (CD45.2^+^), TMCs (CD45.2^+^), and HSCs (CD45.1^+^) in vitro and heterotopically transplanted them to C57BL/7 (CD45.2^+^) mice to explore the feasibility of developing recombinant thymic organoids that can induce T lymphocyte development, in an attempt to educate and generate centrally tolerated donor‐specific T cells, and thus promote transplantation immune tolerance.

## 2 Materials and Methods

### 2.1 Animal Ethics

The animal experiments were approved by the Laboratory Animal Ethics Committee, Shanghai Ninth People′s Hospital, Shanghai JiaoTong University School of Medicine (No. SH9H‐2021‐A504‐SB), and were performed in line with the experimental animal management and use standards. All mice were anesthetized by intraperitoneal injection of 1% pentobarbital sodium (P3761, Sigmo‐Aldrich, USA), and were euthanized by neck dislocation to alleviate the pain of the animals.

### 2.2 Animal Procurement and Experimental Use Statement

A total of 30 wild‐type C57BL/6 (CD45.2^+^) mice (male, 4–6 weeks old) were purchased from Hangzhou Medical College. Six wild‐type SJL/L (CD45.1^+^) mice (male, 6–8 weeks old) were obtained from Vital River Laboratory Animal Technology Co. Ltd. They were all reared in a pathogen free environment with light/dark cycle and free access to food and water. A total of 12 C57BL/6 (CD45.2^+^) mice were used for TECs and TMCs isolation and culture, and 6 SJL/L (CD45.1^+^) mice were employed for HSCs isolation and culture. The remaining 18 C57BL/6 (CD45.2^+^) mice were utilized for in vivo transplantation of thymic aggregates.

### 2.3 Isolation and Culture of Murine TECs and TMCs

To extract mouse TECs, the thymus was removed from euthanized wild‐type C57BL/6 (CD45.2^+^) mice, and the fascia was peeled off and washed with phosphate buffer saline (PBS) (PB180327, Pricella, China) containing Penicillin‐Streptomycin (P/S) solution (PB180327A, Pricella, China). The tissue was cut, digested in 0.125% collagenase I (SCR103, Sigma, Germany) for 1.5 h, blown for 5 min, and left to stand, followed by removal of supernatant. A total of 100 mesh cell sieve (352360, Corning, USA) was added to PBS after blowing, and centrifuged at 1200 rpm for 5 min. After the supernatant was removed, TECs were added to the complete culture medium for 1.5 h, and centrifuged at 1200 rpm for 5 min. Next, TECs were completely suspended in the culture medium, transferred to a 25 T culture bottle, and cultured in an incubator (Forma Steri‐Cult, ThermoFisher, USA) at 37°C for 48 h with the culture medium refreshed. A total of 50 mL TEC complete culture medium consisted of 48.3325 mL dulbecco′s modified eagle medium (DMEM/F12) cell medium (11320033, ThermoFisher, USA), 0.5 mL fetal bovine serum (FBS) (A5670701, Gibco, USA), 0.5 mL P/S solution (PB180120, Pricella, China), 0.5 mL l‐glutamine (ST083, Beyotime, China), 50 μL cholera toxin (HY‐P1446, MCE, USA), 15 μL bovine insulin (HY‐P1156, MCE, USA), 100 μL epidermal growth factor (EGF) (HY‐P7067, MCE, USA), and 2.5 μL hydrocortisone (HY‐N0583, MCE, USA).

To isolate mouse TMCs, the thymus was removed from euthanized wild‐type C57BL/6 (CD45.2^+^) mice. The fascia was peeled off, washed with PBS containing P/S solution, cut, digested with 4% collagenase IV (HY‐E70005D, MCE, USA) for 1 h, blown with PBS, filtered by 100‐mesh cell sieve, and centrifuged at 1200 rpm for 5 min. After the supernatant was removed, the TMCs were completely suspended in medium, transferred to 25 T culture bottle and cultured in a 37°C incubator for 96 h with the culture medium replaced. The unadherent T cells were discarded, and each 50 mL TMC complete medium consisted of 44.5 mL DMEM cell medium (12491015, Gibco, USA), 5 mL FBS, and 0.5 mL P/S solution.

### 2.4 Isolation and Culture of Mouse Bone Marrow HSCs

Wild‐type SJL/L (CD45.1^+^) mice were killed to remove tibia, femur and epiphysis, and the bone marrow was rinsed with DMEM solution containing 1 mL sterile syringe until the marrow cavity turned white. The DMEM solution was inhaled into a centrifuge tube, and centrifuged at 1000 rpm for 5 min. After the removal of supernatant, the cells were precipitated with PBS. The cell suspension was slowly added into a 15 mL centrifuge tube pre‐loaded with 5 mL lymphocyte separation solution (P8860, Solarbio, China) and centrifuged at 2000 rpm for 20 min. The middle bone marrow stromal cell layer (albumen membrane) was absorbed and washed with PBS containing 5% FBS three times. After centrifugation at 1500 rpm for 5 min, the supernatant was discarded, and the bone marrow HSCs were completely suspended in the medium for 2 h and centrifuged at 1500 rpm for 5 min. The supernatant was removed, and the bone marrow HSCs were completely suspended in the medium. The cells were transferred to the 25 T culture bottle and cultured in an incubator at 37°C. 50 mL bone marrow HSCs complete culture medium consisted of 44.5 mL DMEM cell medium, 5 mL FBS, and 0.5 mL diamond‐antibody.

### 2.5 Culture and Transplantation of Recombinant Thymic Organoids

A total of 1 × 10^5^ TECs (including mTECs and cTECs), 5 × 10^4^ TMCs, and 2 × 10^5^ HSCs (TECs:TMCs:HSCs = 2:1:4) were mixed in PBS and centrifuged 1300 rpm for 5 min [17]. After the supernatant was removed, coculture was performed in complete medium of DMEM. A total of 5 ng/mL fms‐related tyrosine kinase 3 ligand (FLT3‐L) (CSB‐BP008733HU1, CUSABIO, China), 5 ng/mL recombinant mouse interleukin 7 (M21610, AbMole, USA) and 1 ng/mL stem cell cytokine (MA0602, meilunbio, China) were added to the DMEM complete medium.

In vivo, the thymus aggregates were cultured on the nuclear pore membrane for 24 h and then implanted to the inguinal region of C57BL/6 (CD45.2^+^) mice. Specifically, An inch‐long incision was made at the midline in the inguinal region. Blunt dissection proceeded to subcutaneous superficial fascia, inguinal fat pad, and then witnessed the femoral vessels. Thymus aggregate was removed with integrity from the nuclear pore membrane, and gently implanted in the groin next to the femoral artery. After implantation, the wound was interruptedly closed using 6‐0 nylon sutures. At 2, 4, and 6 weeks after implantation, the mice were killed. Thymic aggregates were collected and analyzed using flow cytometry, immunofluorescence, and Western blot.

### 2.6 Bioinformatics Analysis

Firstly, we first download microarray data GSE150059 on T‐cell‐mediated acute immune rejection in cardiac allogeneic complex tissue transplantation from gene expression omnibus (GEO) database (https://www.ncbi.nlm.nih.gov/geo). Then differentially expressed genes (DEGs) were screened by DEGs analysis, and cell‐type identification by estimating relative subsets of RNA Transcripts (CIBERSORT) (https://cibersortx.stanford.edu) method was used to calculate the relative content of immune cells in samples. Then, the gene modules highly correlated with damage grade, rejection grade and multiple T cells contents were screened by weighted correlation network analysis (WGCNA), and the corresponding module genes were enriched by Kyoto Encyclopedia of Genes and Genomes (KEGG) and Gene Ontology (GO) analysis. To select genes that could be used in mouse experiments, the Uniprot homology comparison tool was used to check the conserved nature of gene evolution using identity. Finally, interferon gamma (IFNG), interleukin 2 receptor subunit gamma (IL2RG), Fc gamma receptor Ⅲa (FCGR3A), and intercellular cell adhesion molecule‐1 (ICAM1) were selected from the core genes through manual screening.

### 2.7 Identification of TECs, TMCs, and HSCs

The morphology of TECs and TMCs was observed under microscope (×100) (DMi8, Leica, Germany). TECs, TMCs, and HSCs markers were identified using flow cytometer (BD Biosciences, USA). The cells were digested, centrifuged, and re‐suspended with 1× Binding Buffer (BL1890A, Biosharp, China) to obtain cell concentration at 1 × 10^6^/mL. A total of 100 μL cell suspension was placed into 2 mL EP tube. Negative treatment was performed without other treatment. Monopositive tube: TECs were added with 1‐Test protein tyrosine phosphatase, leukocyte common antigen (CD45), epithelial cell adhesion molecule (EpCAM), glutamyl aminopeptidase (Ly‐51), and UEA1 flow antibody, respectively; TMCs were reacted with 1 Test CD45, EpCAM, and endoglin (CD105) flow antibody, respectively; HSCs were mixed with 1 Test lineage cocktail (Lin), stem cell antigen 1 (Sca‐1), and CD117 (c‐Kit) flow antibody, and incubated at 4°C for 30 min in the dark. Sample tube: TECs in cTEC sample tube were added with 1 Test CD45, EpCAM, and Ly‐51 antibodies, while those in mTEC sample tube were added with 1 Test CD45, EpCAM, and *ulex europaeus* agglutinin 1 (UEA1) flow cytometry antibodies; TMCs were incubated with 1 Test CD45, EpCAM, and CD105 antibodies; HSCs were cultured with 1 Test Lin, Sca‐1 and c‐Kit antibodies at 4°C for 30 min without light. The negative control, single positive tube and sample tube were added with 400 μL PBS, and centrifuged at 600*g* for 5 min. The supernatant was removed, and the cells in suspension were added with 400 μL PBS containing 0.3% bovine serum albumin (BSA) (ST023, Beyotime, China). The experiment was repeated three times. Antibodies used in this experiment included CD45 (MHCD4530, ThermoFisher, USA), EpCAM (14‐9326‐82, ThermoFisher, USA), Ly‐51 (740025, BD Biocsiences, USA), UEA1 (L32476, ThermoFisher, USA), CD105 (17‐1057‐42, ThermoFisher, USA), Lin (88‐7772‐72, ThermoFisher, USA), Sca‐1 (12‐5981‐82, ThermoFisher, USA), and c‐Kit (17‐1171‐82, ThermoFisher, USA).

### 2.8 Flow Cytometry

After 2, 4, and 6 weeks of transplantation, mouse thymus aggregates were collected, and the single‐cell suspensions were prepared and then washed with dye buffers. Cells were stained with antibodies, CD4 (25‐0049‐42, ThermoFisher, USA), CD3 (14‐0037‐82, ThermoFisher, USA), CD8 (12‐0088‐42, ThermoFisher, USA), forkhead box P3 (FOXP3, 11‐4777‐42, ThermoFisher, USA), CD45.1 (17‐0453‐82, ThermoFisher, USA) and CD45.2 (12‐0454‐82, ThermoFisher, USA) for 30 min at 4°C. Subsequently, analysis was performed using FACSCalibur flow cytometry (BD Biosciences, USA) and FlowJo v.10 software (FlowJo, USA). The quantity and proportion of CD4^−^ CD8^−^ (double negative, DN), CD4^+^ CD8^+^ (double positive, DP), CD3^+^ CD4^+^ CD8^−^ (single positive, SPCD4), CD3^+^ CD8^+^ CD4^−^ (SPCD8), CD4^+^ FOXP3^+^, CD8^+^ FOXP3^+^ (Treg), and CD45.1/CD45.2 cells were detected. Negative cell clusters were used as the negative control to establish gates.

### 2.9 Immunofluorescence

The collected thymus aggregates were fixed with fixative solution (P0098, Beyotime, China) for 30 min, and incubated with Alexa Fluor 555 labeled cytokeratin 14 (CK14, ab214391, Abcam, UK) and Alexa Fluor 488 labeled CK8 (53‐9003‐82, ThermoFisher, USA) antibodies. The nucleus was stained with 2‐(4‐Amidinophenyl)‐6‐indolecarbamidinedihydrochloride (DAPI) dye solution (C1005, Beyotime, China). Then the expressions of CK14 and CK8 were observed under fluorescence microscope (×400) (Ni‐U, NIKON, Japan).

### 2.10 Western Blot

The total protein of thymus aggregates was extracted from RIPA lysate (R0010, Solarbio, China), and measured using the BCA Protein Assay Kit (BCA1, Sigma‐Aldrich, USA). After sample loading, electrophoresis and membrane transfer, membranes were blocked using 5% skim milk powder (1.15363, Millipore, USA) and then probed overnight at 4°C with primary antibodies, IFNG (0.1 μg/mL, 19 kDa, 500‐P119‐1MG, ThermoFisher, USA), IL2RG (CD132, 1:500, 52 kDa, PA5‐80730, ThermoFisher, USA), FCGR3A (CD16, 1:500, 32 kDa, PA5‐106915, ThermoFisher, USA), and ICAM1 (CD54, 1 μg/mL, 50 kDa, 701254, ThermoFisher, USA). Then membranes were cultured with horseradish peroxidase‐labeled goat anti‐rabbit IgG (ab6721, Abcam, UK, 1:2000) at 37°C for 1 h, followed by color rendering by chemiluminescence method and exposure in the dark. Absorbance values of target proteins were obtained by scanning with image analysis software, using glyceraldehyde‐3‐phosphate dehydrogenase (GAPDH) (1:1000, 32 kDa, #2118, CST, USA) as the internal reference.

### 2.11 Statistical Analysis

Statistical analysis was carried out using GraphPad 8.0, and the measurement data were expressed by mean ± standard deviation. Tukey′s post hoc test and one‐way ANOVA were utilized for comparisons among multiple groups, while the independent sample *t* test was employed to analyze data between two groups. *p* < 0.05 implied statistically significant difference.

## 3 Results

### 3.1 Identification of TECs, TMCs, and HSCs

**Figure 1:**
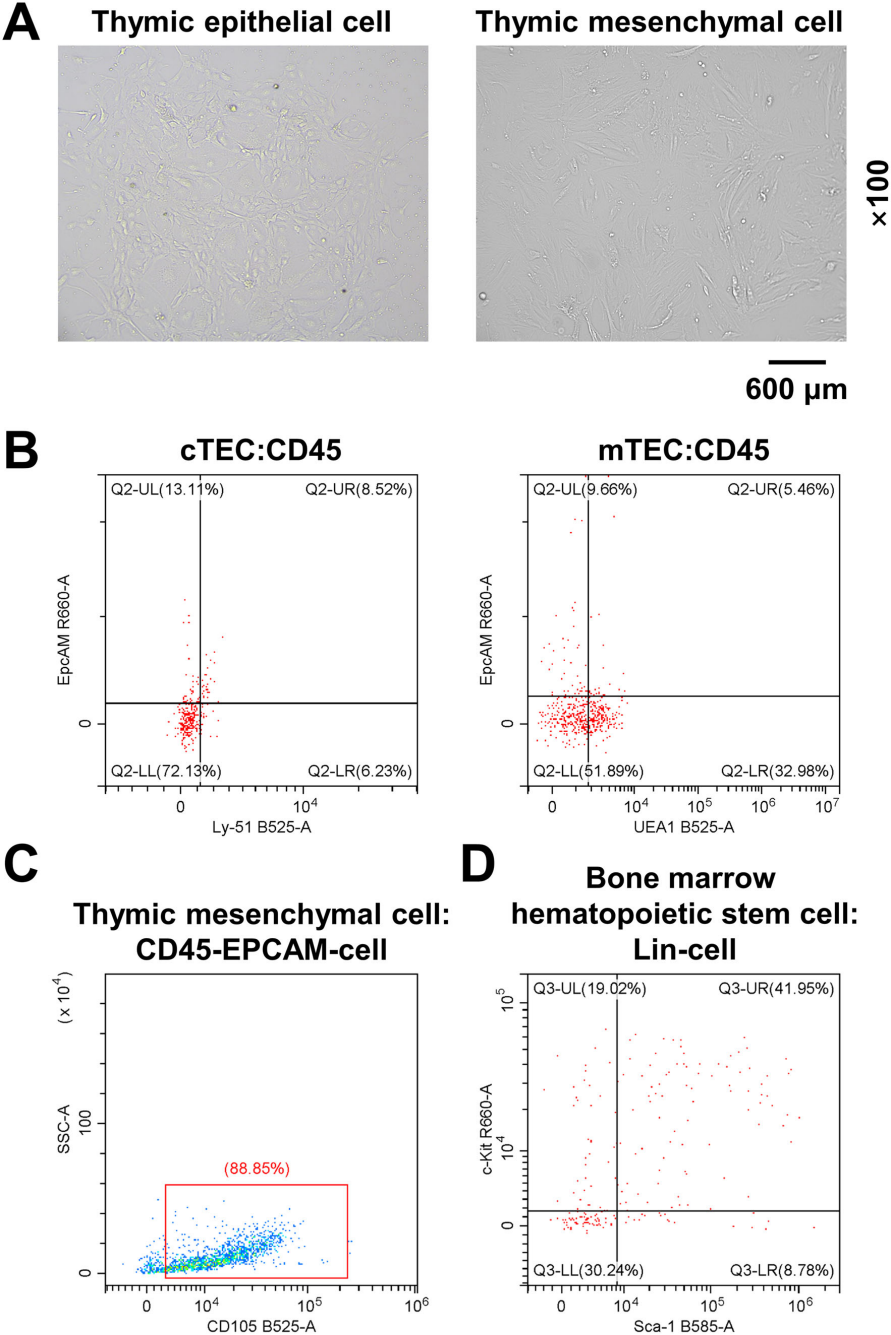
Cell culture and identification. (A) Morphological characteristics of thymic epithelial cells (TECs) and thymic mesenchymal cells (TMCs) in mice (×100). (B) Mouse TEC markers were detected by flow cytometry. (C) Flow cytometry was used to detect mouse TM markers. (D) Flow cytometry was used to detect bone marrow hematopoietic stem cells (HSCs).

TECs and TMCs were isolated and cultured from mice thymus. The isolated TECs adhered to the wall slowly, and a small number of round cells adhered to the petri dish for 24 h. Four days later, the cell adhesion was relatively complete, with round, polygon and dendritic adherent T cells continuously generated from its edge. After 10 days of culture, the cells grew rapidly and a large number of adherent T cells appeared. Following 25 days of culture, a large number of cells fused and showed a typical pebble‐like arrangement (Figure 1A). The isolated TMCs adhered rapidly and were arranged neatly in a long spindle shape under an inverted microscope at Day 5. The cell proliferation rate was higher and cell fusion was observed at Day 10 (Figure 1A).

Moreover, isolation, and identification of TECs, TMCs, and HSCs by in vitro sorting using flow cytometry. TECs were labeled with CD45, EpcAM, Ly‐51, and UEA1 antibodies, and the flow cytometry detection results showed negative expression of CD45 and positive expressions of EpcAM, Ly‐51, and UEA1 (Figure 1B). The double positive rate of EpcAM and Ly‐51 was 8.52%, and the double positive rate of EpcAM and UEA1 was 5.46% (Figure 1B). After TMCs were evolved to the third generation, CD45, EpcAM, and CD105 antibodies were labeled, and the flow cytometry results showed that CD45 and EpcAM were negatively expressed, while CD105 was positively expressed on the cell surface. The positive rate of CD105 was 88.85% (Figure 1C). HSCs were labeled with Lin, Sca‐1 and c‐Kit antibodies, and Lin was negatively expressed on the cell surface, while Sca‐1 and c‐Kit were positively expressed. The double positive rate of Sca‐1 and c‐Kit was 41.95% (Figure 1D). The above results indicated that the TECs, TMCs, and HSCs isolated and cultured had typical characteristics and expressed unique markers.

### 3.2 Results of Bioinformatics Analysis

**Figure 2:**
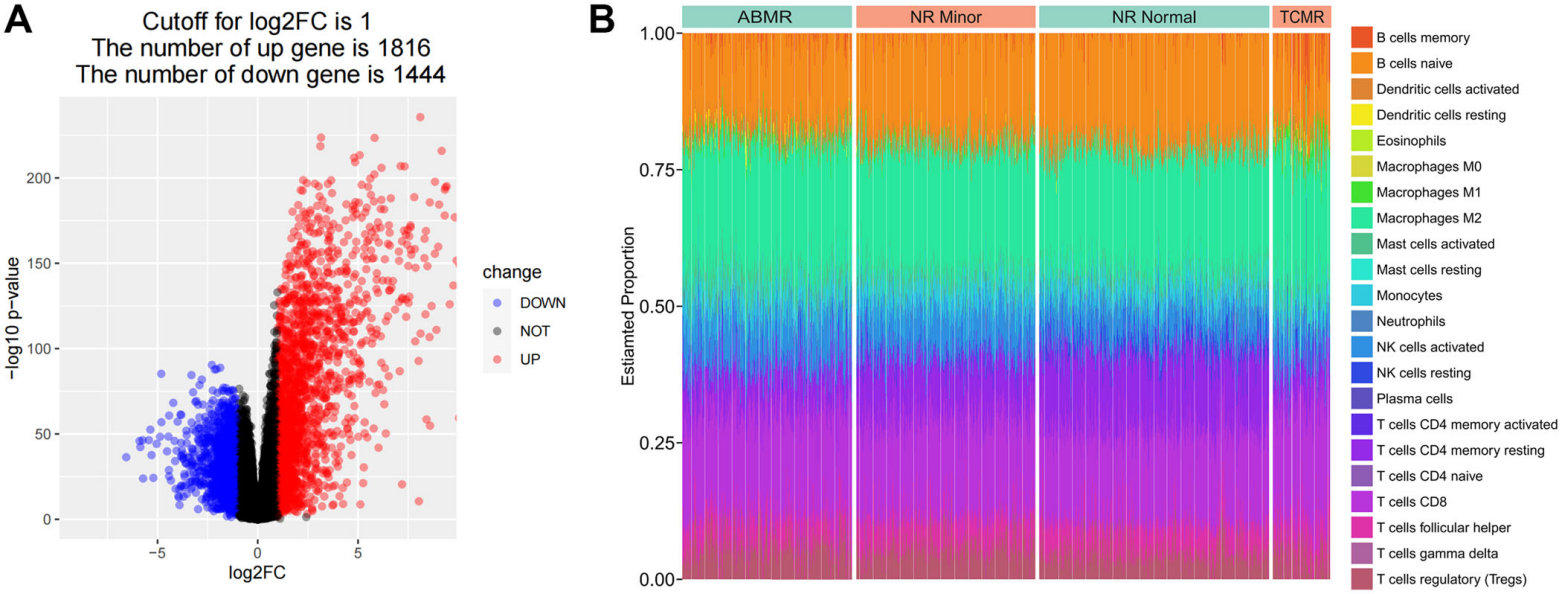
Differential expression analysis and construction of sample feature matrix. (A) R‐packet limma was used for differential expression analysis, and 3260 genes with *p*.adj < 0.05 and log2FC > 1 were considered as differentially expressed genes. (B) Cell‐type identification by estimating relative subsets of RNA Transcripts (CIBERSORT) was performed to calculate the relative immune cell content.

Moreover, this study analyzes and screens genes related to T cell development and immune tolerance. R‐packet limma was used for differential expression analysis, and the volcano map was shown in Figure 2A. A total of 3260 genes that met *p*.adj < 0.05 and log2FC > 1 was regarded as DEGs (Figure 2A).

Then, CIBERSORT method was used to calculate the content of immune cells, and the relative content of immune cells in samples was shown in Figure 2B.

**Figure 3:**
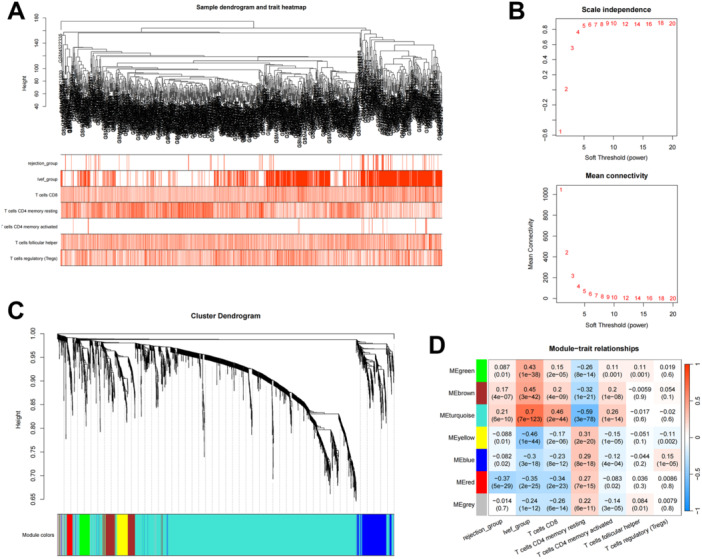
Weighted correlation network analysis (WGCNA) analysis. (A) The expression matrix of samples was clustered and outlier samples were removed. (B) Construction of scale‐free networks (power: 1–20). (C) The WGCNA was constructed, and 7 gene modules were clustered and analyzed. (D) Calculation of the relationship between 7 color blocks and sample characteristics.

Moreover, to screen genes mediated by T cells that are associated with immune rejection and degree of damage, a sample signature matrix was constructed for subsequent WGCNA analysis. The expression matrix of samples was clustered and outlier samples were removed (Figure 3A). According to Figure 3B, the power value was selected as 6. Therefore, select 6 as the optimal soft threshold for subsequent analysis. The coexpression network is constructed based on the optimal soft threshold, and the gene cluster tree can be drawn after the gene is divided into different modules (Figure 3C). Then, the relationship between 7 color blocks and sample features was determined (Figure 3D). We noted that 322 genes in the MEturquoise module (Table S1) were highly positively correlated with rejection and damage related features, positively correlated with CD8^+^ T cells, regulatory T cells, and follicular helper T cells, and highly negatively correlated with dormant CD4^+^ T memory cells. Therefore, it is speculated that the genes of this module may play an important role in the process of transplant rejection or tissue damage.

**Figure 4:**
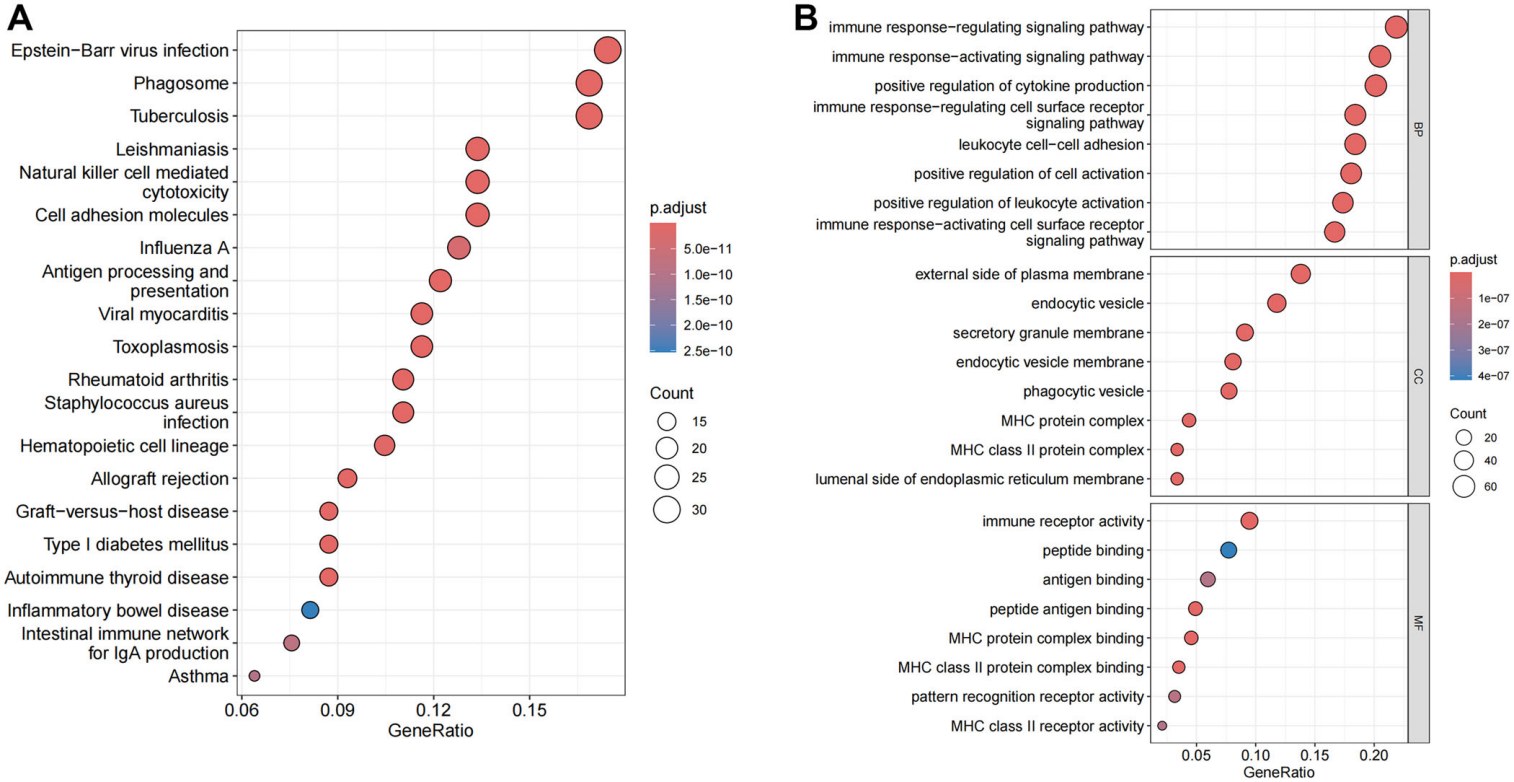
Selection of candidate genes. (A) Kyoto encyclopedia of genes and genomes (KEGG) enrichment results of 322 core gene of blue module. (B) Gene Ontology (GO) enrichment results of 322 core gene of blue module.

Finally, 322 core genes in the MEturquoise module were performed by GO and KEGG analysis (Tables S2 and S3). The enrichment results are shown in Figure 4, and these genes are highly correlated with immune response function. In the KEGG enrichment results, candidate genes were selected from immune disease and immune system subcategories by artificial screening. Collectively, IFNG, IL2RG, FCGR3A, and ICAM1 associated with immune diseases were selected from the core genes through manual screening.

### 3.3 Detection of Recombinant Thymus Organ‐Associated Proteins

**Figure 5:**
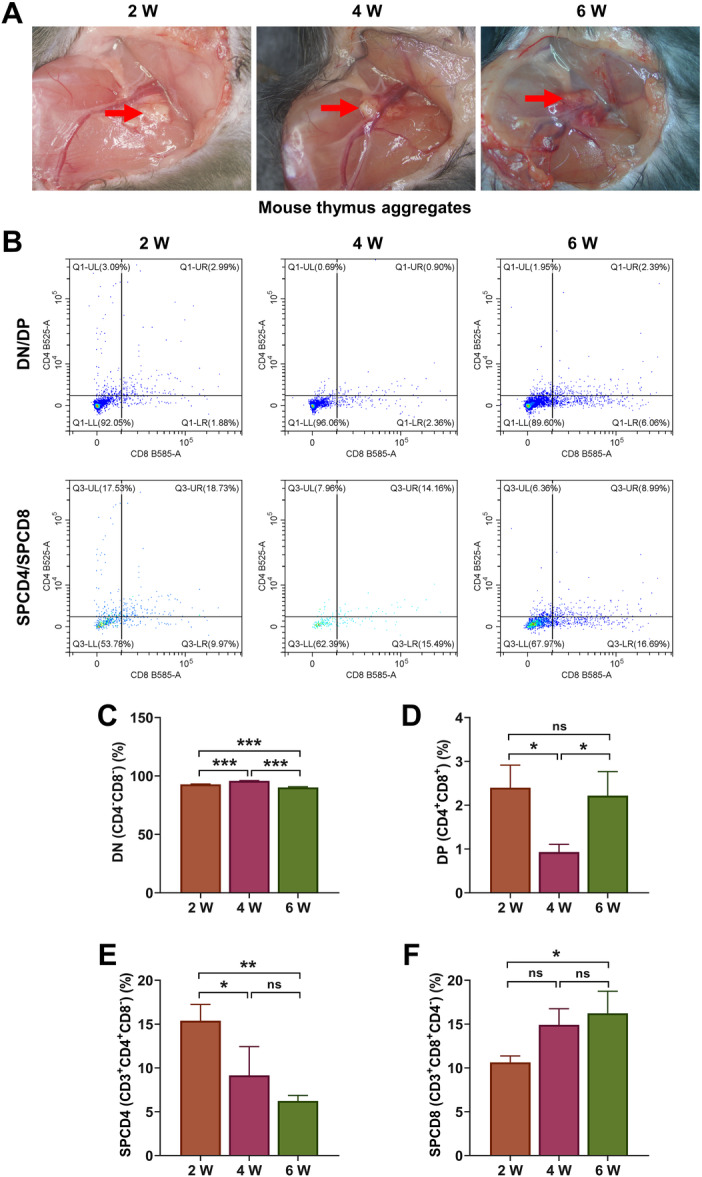
Flow cytometry analysis. (A) Observation of mouse thymus aggregates, red arrow: recombinant thymus organs. (B–F) The percentages of cluster of differentiation (CD4)^−^ CD8^−^ (double negative, DN), CD4^+^ CD8^+^ (double positive, DP), CD3^+^ CD4^+^ CD8^−^ (single positive, SPCD4), and CD3^+^ CD8^+^ CD4^−^ (SPCD8) T cells were detected by flow cytometry. All experiments were repeated three times. *p < 0.05, **p < 0.01, ***p < 0.001.

Moreover, to demonstrate the ability of thymic aggregates to support T cells in vivo, thymic aggregates containing TECs, TMCs, and HSCs were implanted to the inguinal region of C57BL/6 (CD45.2^+^) mice. Figure 5A indicates the growth of recombinant thymus organs after transplantation into mice at different times.

**Figure 6:**
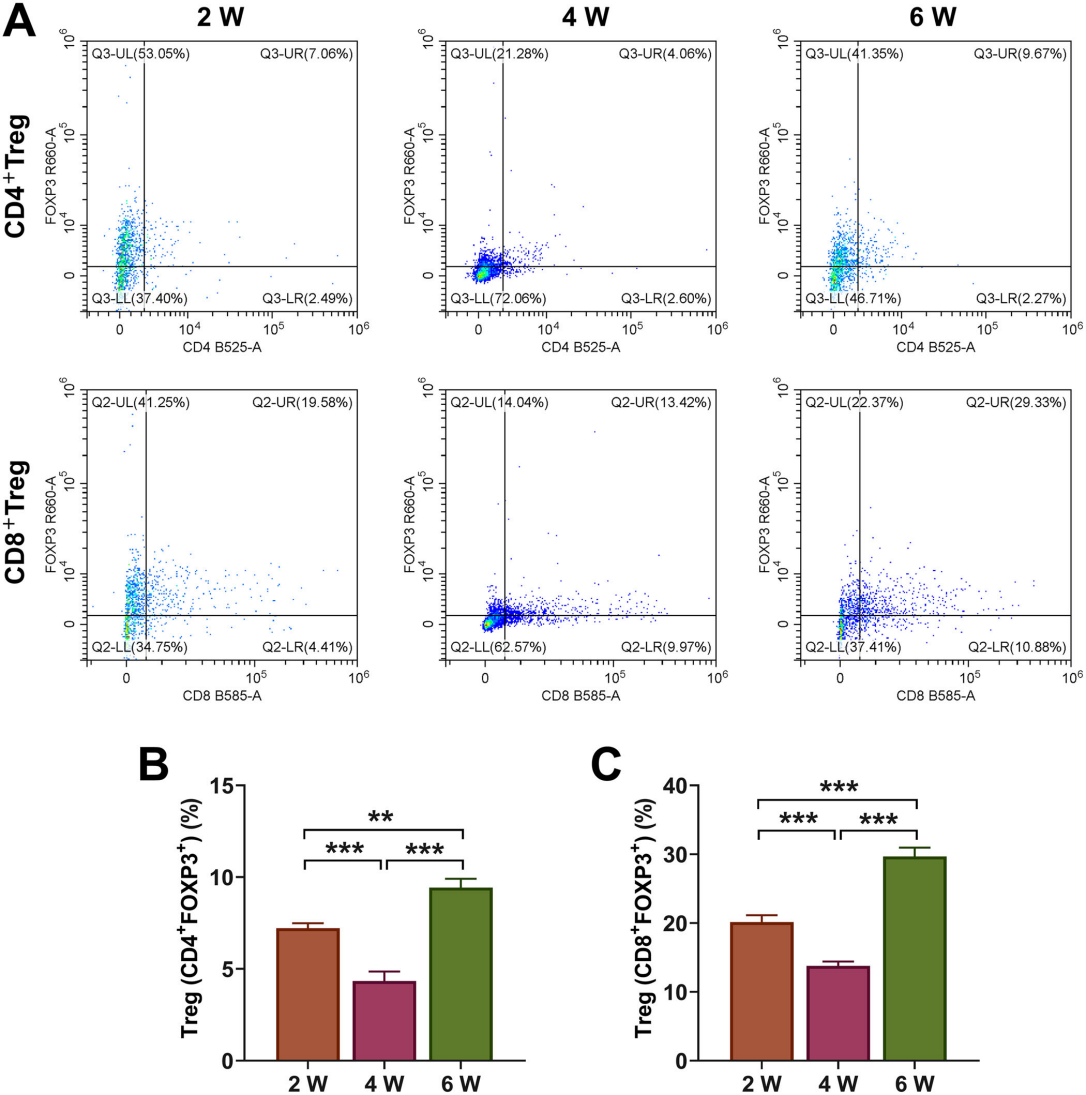
Flow cytometry analysis. (A–C) The percentages of regulatory T cells (Treg), CD4^+^ forkhead box protein P3 (FOXP3)^+^, and Treg (CD8^+^ FOXP3^+^) T cells were detected by flow cytometry. All experiments were repeated three times. **p < 0.01, ***p < 0.001.

**Figure 7:**
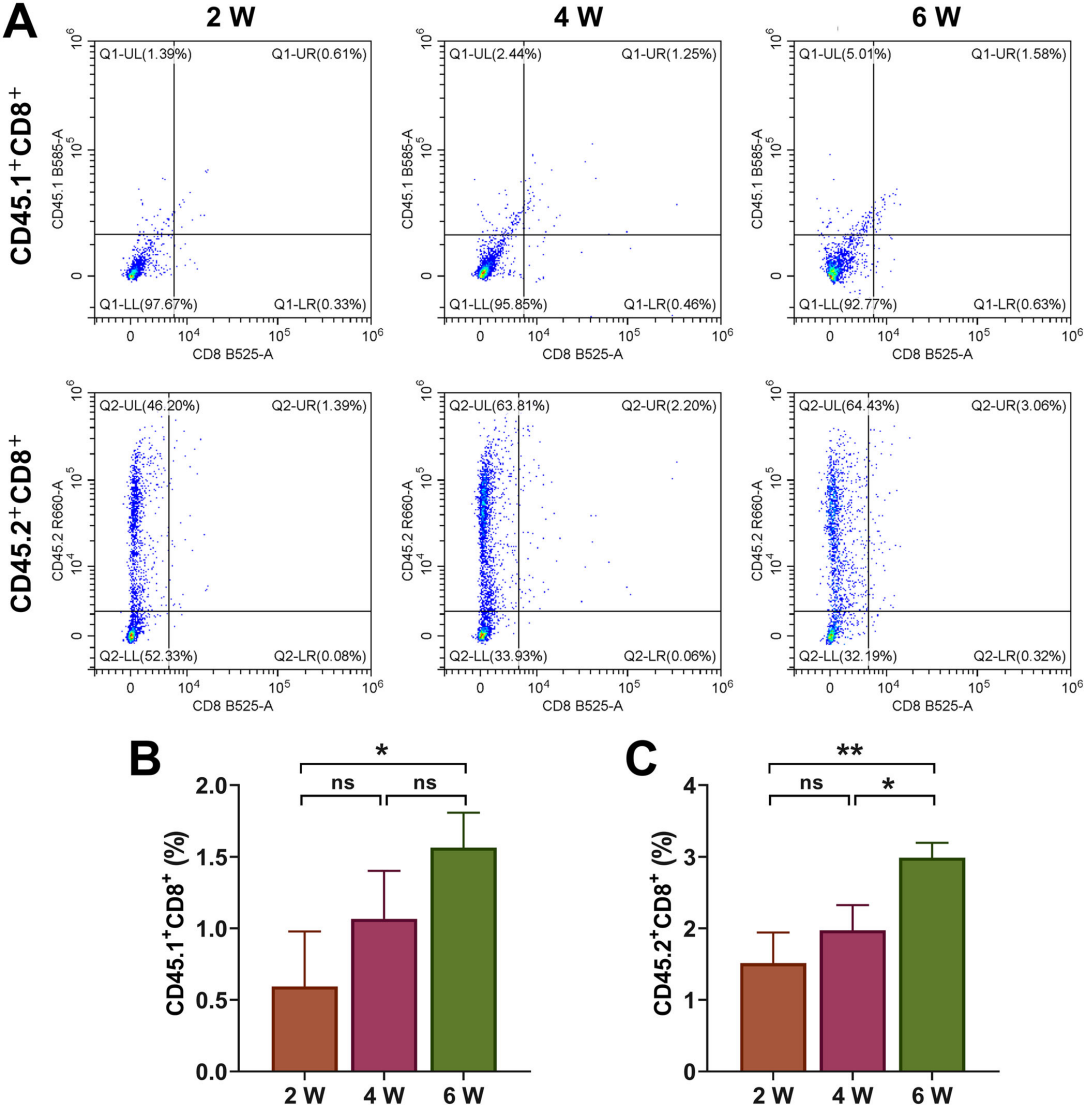
Flow cytometry analysis. (A–C) The percentages of CD45.1^+^ CD8^+^ and CD45.2^+^ CD8^+^ T cells were tested by flow cytometry. All experiments were repeated three times. *p < 0.05, **p < 0.01.

**Figure 8:**
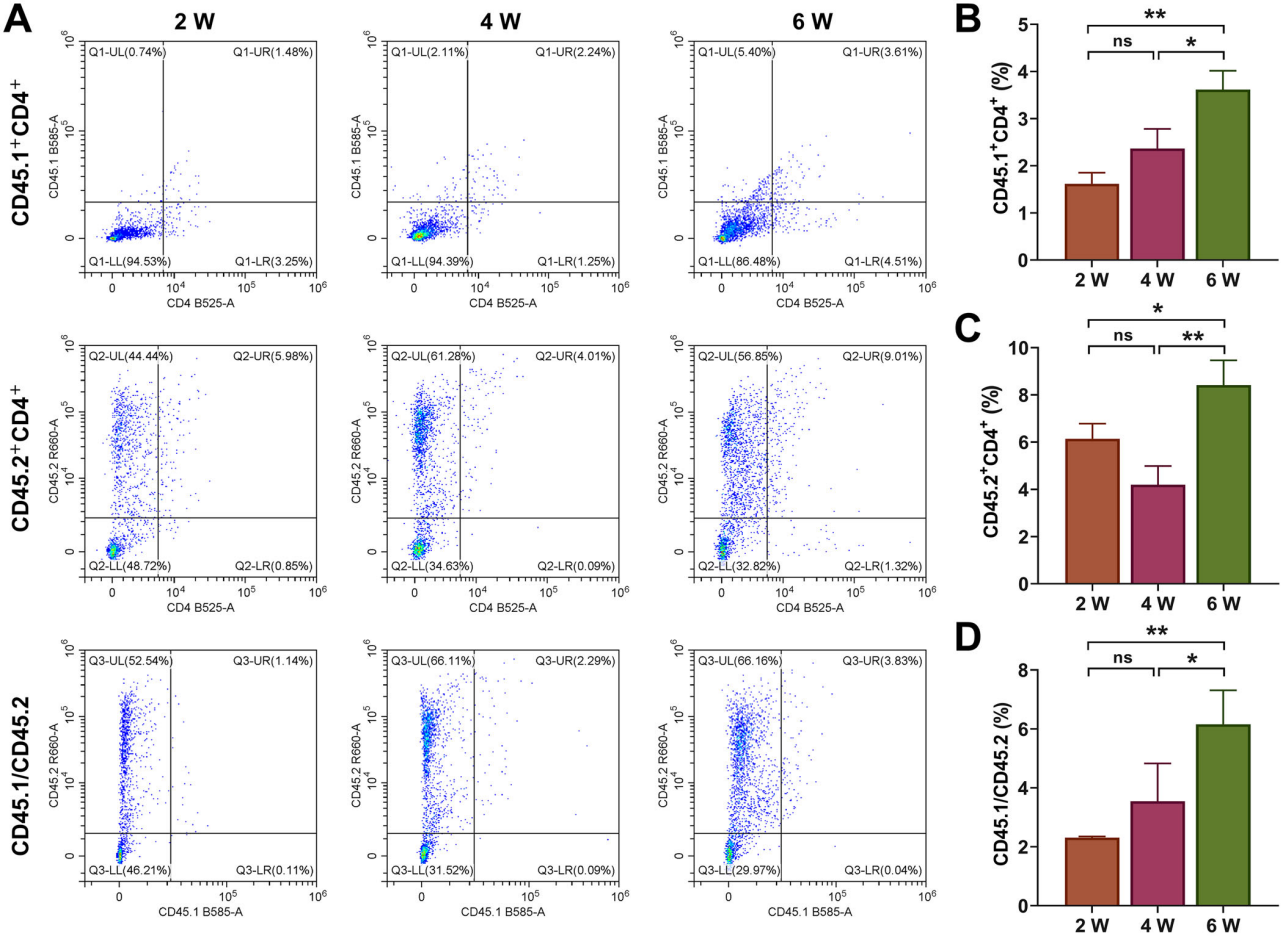
Flow cytometry analysis. (A–D) The percentages of CD45.1^+^ CD4^+^, CD45.2^+^ CD4^+^ T, and CD45.1/CD45.2 cells were determined by flow cytometry. All experiments were repeated three times. *p < 0.05, **p < 0.01.

After the recombinant thymic organoids were implanted into mice for 2/4/6 weeks, we examined the production of different T cells, including CD4^−^ CD8^−^ (DN), CD4^+^ CD8^+^ (DP), CD3^+^ CD4^+^ CD8^−^ (SPCD4), CD3^+^ CD8^+^ CD4^−^ (SPCD8), Treg (CD4^+^ FOXP3^+^), Treg (CD8^+^ FOXP3^+^), CD45.1^+^ CD4^+^, CD45.2^+^ CD4^+^, CD45.1^+^ CD8^+^, CD45.2^+^ CD8^+^ T, and CD45.1/CD45.2 cells. First, as shown in Figure 5, compared with the results at Week 2, the number of DN cells was increased at Week 4, but decreased at Week 6 (Figure 5B,C, *p* < 0.01), and the number of SPCD4 continued to reduce (Figure 5B,E, *p* < 0.05). The number of DP was also diminished markedly at Week 4 relative to Week 2, but elevated significantly at Week 6 compared to Week 4 (Figure 5B,D, *p* < 0.05). However, the amount of SPCD8 continued to increase over time, with the amount significantly higher at Week 6 than Week 2 (Figure 5B,F, *p* < 0.05). It was worth noting that Treg cells were decreased evidently at Week 4, but increased significantly at Week 6 (Figure 6A–C, *p* < 0.01). There was no significant change in the number of CD45.1^+^ CD8^+^ and CD45.2^+^ CD8^+^ cells at Week 2 and Week 4 (Figure 7A–C), the number of CD45.1^+^ CD8^+^ was increased at Week 6 (Figure 7A–C, *p* < 0.05). The number of CD45.1^+^ CD4^+^ cells was increased significantly at Week 6 (Figure 8A,B, *p* < 0.05), and the number of CD45.2^+^ CD4^+^ cells at Week 4 was less than that at Week 2, but the number at Week 6 was more than that at Week 4 (Figure 8A,C, *p* < 0.05). Finally, we found that the percentages of CD45.1/CD45.2 cells at Week 6 were also higher than at Week 2 (Figure 8A,D, *p* < 0.01).

**Figure 9:**
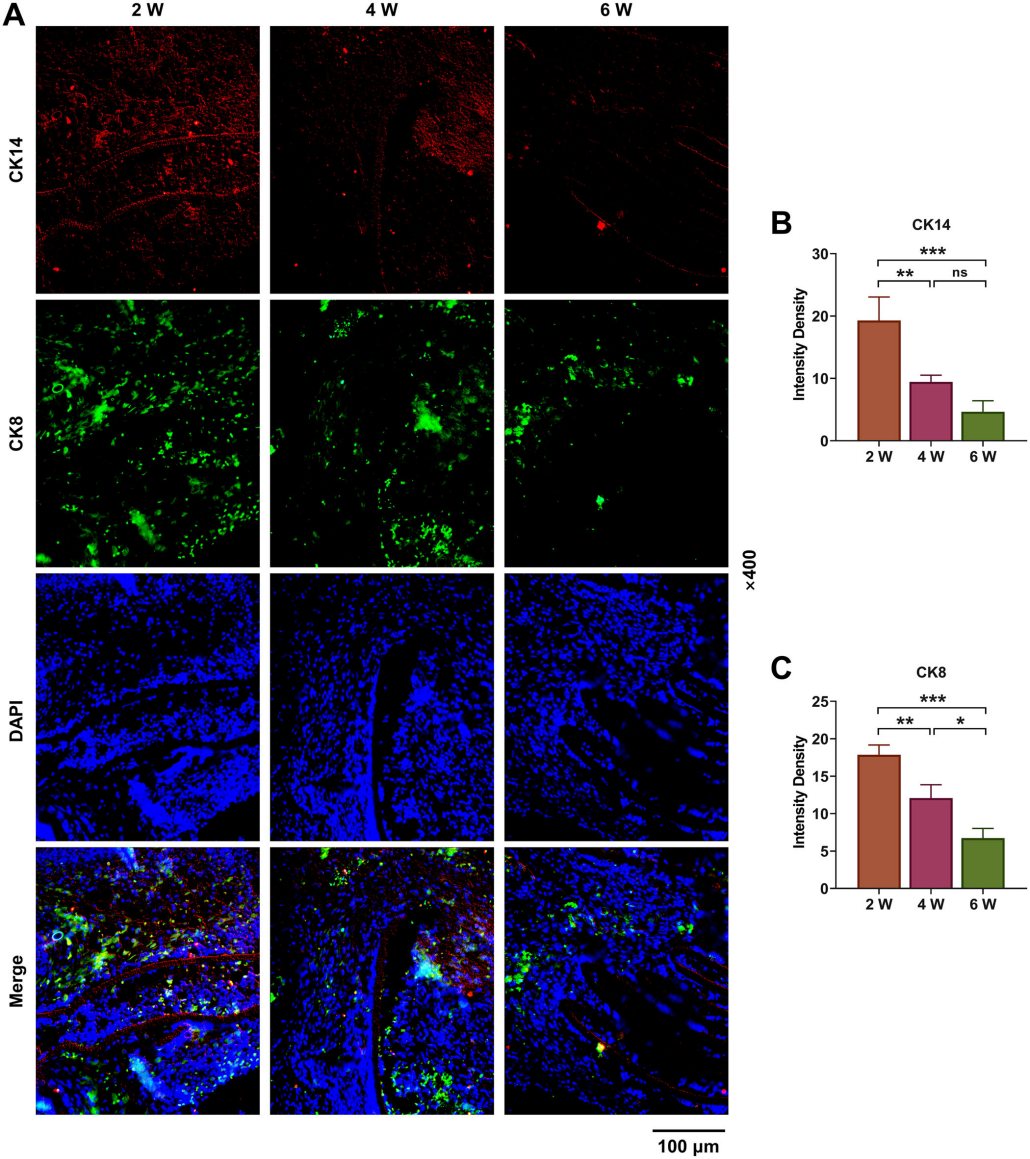
Immunofluorescence analysis. (A–C) The expression levels of cytokeratin 14 (CK14) and cytokeratin 8 (CK8) were detected with prolonged differentiation time by immunofluorescence (×400). All experiments were repeated three times. *p < 0.05, **p < 0.01, ***p < 0.001.

**Figure 10:**
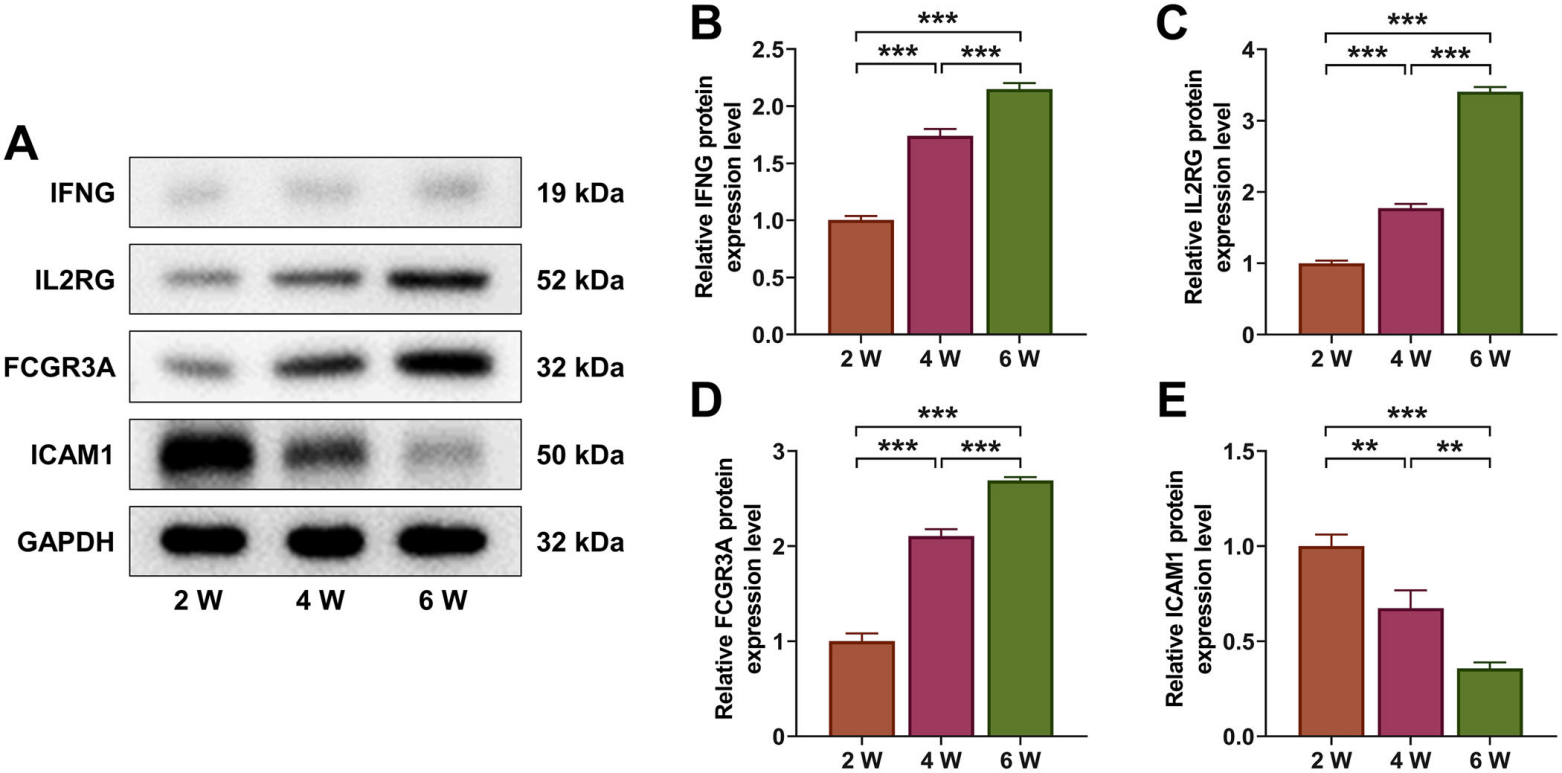
Western blot analysis. (A–E) Protein levels of interferon gamma (IFNG), interleukin 2 receptor subunit gamma (IL2RG), Fc gamma receptor Ⅲa (FCGR3A) and intercellular cell adhesion molecule‐1 (ICAM1) were measured by Western blot assay, with glyceraldehyde‐3‐phosphate dehydrogenase (GAPDH) as the internal parameter. All experiments were repeated three times. **p < 0.01, ***p < 0.001.

Further, T‐cell development in the thymus depends mainly on sequential interactions between the thymocytes and this distinct microenvironment in cortex and medulla of the thymus [18]. Immunofluorescence results confirmed that with the extension of culture time, the protein levels of CK14 (medullary) and CK8 (thymic cortical) were significantly decreased (Figure 9A–C, *p* < 0.01). Finally, protein levels of INFG, IL2RG, FCGR3A, and ICAM1 were detected by Western blot assay. The results showed that protein levels of INFG, IL2RG, and FCGR3A associated with immune diseases were evidently upregulated (Figure 10A–D, *p* < 0.001), while CAM1 protein levels were significantly downregulated as the differentiation time prolonged (Figure 10A,E, *p* < 0.01). These results indicated that although the recombinant thymus organs we cultured produced different types of T cells, regulated the immune disease‐related genes, demonstrating the possibility of inducing T cells bank production in vitro, the number of certain types of T cells produced was not stable enough.

## 4 Discussion

Various VCA, such as facial, limb and abdominal wall transplantation, has been performed worldwide in clinic [19]. The administration of immunosuppressants or immunomodulators is unavoidable to mitigate or prevent rejection [19]. However, a plethora of inevitable complications, including opportunistic infection and secondary malignancies, occur due to long‐term use of immunosuppressive medication [20]. Therefore, researchers have shifted their focus to cell therapy as a preventative measure against immunological rejection.

In our study, HSCs, TECs, and TMCs were cocultured and implanted into C57BL/7 (CD45.2^+^) mice to induce the generation of a recombinant thymic organoid. T cell production at different time intervals post‐transplantation was evaluated using flow cytometry, and juxtaposed with the pre‐transplantation period [12]. Through bioinformatics analysis, the core genes INFG, IL2RG, FCGR3A, and ICAM1 associated with immune diseases were screened and their expressions were also measured at the protein level. INFG, which is primarily released by T cells and properties, and is necessary for regular immunological surveillance [21]. The glycoprotein IL2RG is expressed on the surface of most lymphocytes and is a frequent subunit of numerous significant immune factors [22]. Many immunological effector cells produce FCGR3A, a transmembrane protein that belongs to the immunoglobulin superfamily [23]. ICAM1 can effectively mediate the cell‐to‐cell adhesion response, with low expressions in various cells, including endothelial cells, immune cells, and certain epithelial cells, and is upregulated upon inflammatory stimuli [24]. Herein, the upregulation of INFG, IL2RG and FCGR3A and the downregulation of ICAM‐1 indicated that the differentiation‐induced recombinant thymus organs had markers related to immune regulation.

The role of TMCs and TECs in T cell development has been explored through recombinant thymus organ culture, and the results showed that TMCs mainly influence the early stage of T cell development, while TECs impact the later stage, including positive and negative selection [25]. T cells are a vital component of the body′s adaptive immunity and are crucial in combating tumor cells. The thymus supports T cells differentiation, proliferation, development, and domestication during their normal developmental phase. The development of T cells can be summarized into three stages, namely, T‐cell receptor (TCR) gene recombination, positive selection and negative selection [26]. After entering the thymus, the bone marrow‐derived pro‐T cells first undergo TCR gene recombination, which is accompanied by rapid expansion of T cells and produces a large number of double‐positive T cells with different TCR structures. Then, cTEC and mTEC in the thymic stromal cells are primarily responsible for the positive and negative selection of T cells [27]. cTEC, located in the cortical region of the thymus′ superficial layer, primarily selects double‐positive T cells to acquire major MHC restriction, which maintains the TCR′s functional integrity [28]. The establishment of central immunological tolerance depends on mTEC, located in the deep medullary part of the thymus, that negatively selects single‐positive T cells [28].

Classical T cells (CD4^+^ T, CD8^+^ T, and Tregs) and non‐classical T cells constitute the mature T cell area. Tregs, also referred to as regulatory T cells, are a subpopulation of CD4^+^ T cells, which can suppress immune responses of other cells, maintain the immune system′s capacity to tolerate its own constituents, and preserve immunological homeostasis [29, 30]. The expressions of FOXP3, CD25 and CD4 are the phenotypic characteristics of Tregs. Besides, Tregs play an important role in infection, tumor, organ transplantation, prevention of autoimmune diseases, and maintenance of immune balance [29]. In the establishment of recombinant thymus organs in vitro, the thymus epithelial cell surface antigens, expressions of related functional genes, and T cells receptor phenotypes were examined. The expression levels of CK14 and CK8 were gradually decreased. Different subtypes of T cells were produced by the recombinant thymus organs, among which the number of regulatory T cells CD4^+^ Treg and CD8^+^ Treg was signally higher with the extension of transplantation time than in the pre‐transplantation period. The prominent role of Tregs in maintaining immune homeostasis and organ transplantation suggested that the cultured thymic organoids may have the ability of immune tolerance after transplantation and reduce the immune response of xenotransplantation. However, previous study catch our eyes, it was noted that memory‐like CD127 Tregs develop in lymphoid organs. They are further reprogrammed within the graft and have an established phenotype as a memory‐like CD127 tissue Treg with a unique molecular signature, which is critical for maintaining tolerance [31]. Moreover, both CD25^+^FOXP3^−^ Treg and CD25^−^Foxp3^lo^ Treg contributed to mature Treg development in the thymus [32]. Therefore, this remind us that The development and functions of different types of Treg cells vary and are interrelated in different tissue environments, and they jointly participate in the immune regulation process. Therefore, more research are need to further study.

However, there are some limitation in this study. First, although HSCs have been successfully induced to differentiate into recombinant thymus organs, functional experiments in vitro are still needed on T cells, such as using lipopolysaccharide and antigen‐presenting cells to build an immune response environment and explore the function of T cells. Then, CD4^+^/CD8^+^FOXP3 were only selected to define Treg cells. However, the characteristics of Treg cells may be affected by various factors, and the combination of more markers may be able to define Treg cells more accurately and comprehensively. Therefore, more markers (such as CD25, CD127) should be used to further accurately define Treg cells in the future. Moreover, further study with VCA model are warranted to confirm the regulatory role of thymus organs in immune tolerance. A more comprehensive and detailed analysis holds important reference value for the clinical application of cell therapy to induce immune tolerance.

## 5 Conclusions

In conclusion, our study demonstrates that the recombinant thymus organs established by TECs, TMCs, and HSCs produce multiple subtypes of T cells, especially CD4^+^ Treg and CD8^+^ Treg, and can regulate the self‐recognition and tolerance of T cells to tissues or cells derived from donors, promoting the induction and establishment of transplantation immune tolerance.

## Author Contributions

Conceptualization: Xiuxia Wang and Shun Yu. Data curation: Yucheng Qiu, Jun Yang, Fei Liu, and Xianyu Zhou. Writing – original draft: Xiuxia Wang and Shun Yu. Writing – review and editing: Xiuxia Wang and Shun Yu.

## Ethics Statement

The animal experiments were approved by the Laboratory Animal Ethics Committee, Shanghai Ninth People′s Hospital, Shanghai JiaoTong University School of Medicine (No. SH9H‐2021‐A504‐SB), and were performed in line with the experimental animal management and use standards.

## Conflicts of Interest

The authors declare no conflicts of interest.

## Supporting information

Graphical abstract revised.

## Data Availability

The data that support the findings of this study are available from the corresponding author upon reasonable request.
