# Supplementary figures and images for: Development of Thymic Organoids Heterotopically to Educate and Induce T Lymphocytes
Source: Immun Inflamm Dis. 2025 Aug 22;13(8):e70229. doi: 10.1002/iid3.70229 (PMC12371559; doi:10.1002/iid3.70229)

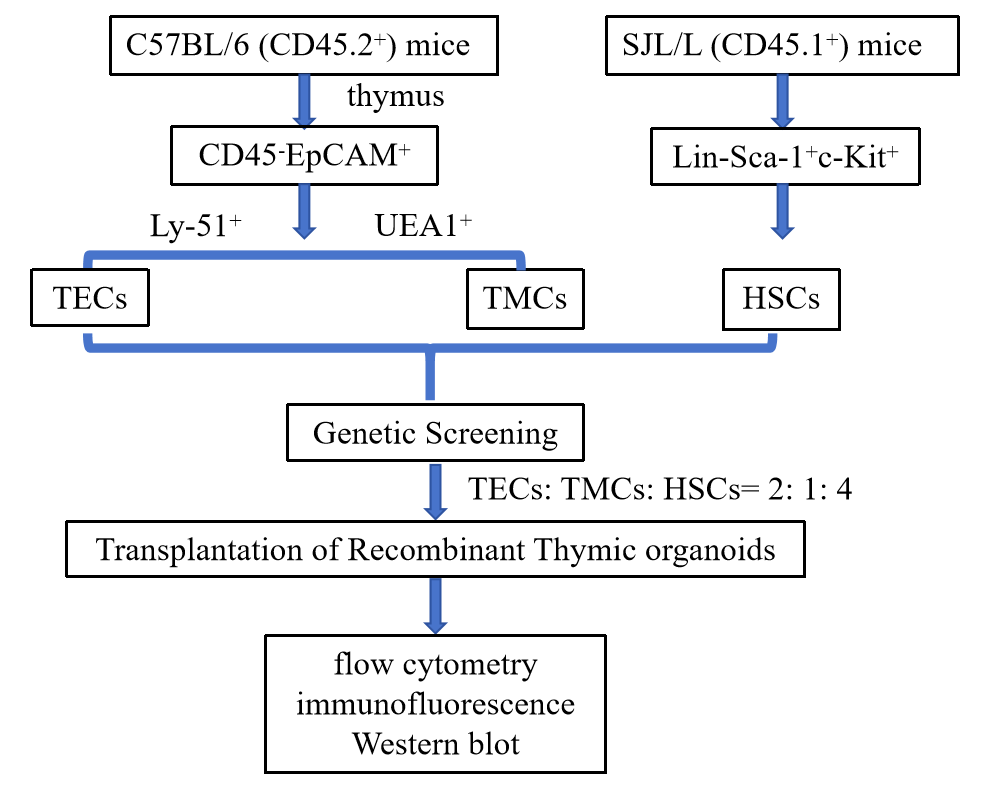

Supplement: Supplementary file 1 — Graphical abstract revised. [file IID3-13-e70229-s001.tif]
